# Assessment of pain intensity after total hip arthroplasty using the Visual Analogue Scale (VAS)

**DOI:** 10.25122/jml-2024-0362

**Published:** 2024-12

**Authors:** Madalin Bulzan, Simona Cavalu, Florica Voiță-Mekereș, Călin Tudor Hozan

**Affiliations:** 1 Faculty of Medicine and Pharmacy, University of Oradea, Oradea, Romania

**Keywords:** pain, total hip arthroplasty, Visual Analogue Scale, analgesics

## Abstract

This study aimed to assess pain intensity in patients who underwent total hip arthroplasty (THA) using the Visual Analogue Scale (VAS). The study was conducted between 2022 and 2023, enrolling 203 patients admitted to the Orthopedics Department. Participants self-reported their post-surgical pain 24 hours after THA by selecting a VAS rating that best represented their personal experience. Based on their diagnosis, participants were categorized into four relatively homogeneous groups: left-sided coxarthrosis, right-sided coxarthrosis, unilateral THA for bilateral coxarthrosis, and bilateral THA for bilateral coxarthrosis. Data were analyzed using SPSS, with frequency analysis performed using the chi-square (χ^2^) test. Pain intensity in patients undergoing uncemented THA ranged from mild irritation to pain requiring moderate analgesics. The statistical analysis revealed significant differences in pain frequencies across groups (χ^2^ (24, 203) = 37.192; *P* = 0.04), with variations largely attributable to the type of THA performed. VAS scores indicated that patients with unilateral THA for coxarthrosis reported moderate pain lasting up to 30 minutes, necessitating moderate-intensity analgesics. In contrast, participants undergoing bilateral THA experienced more severe pain, requiring the administration of strong analgesics for effective pain relief and increased functionality. Among surgical procedures, uncemented total hip prostheses were associated with the highest frequency of manageable pain, characterized as irritation or mild discomfort requiring moderate analgesics.

## INTRODUCTION

Total hip arthroplasty (THA) is one of the most successful elective surgical interventions to alleviate hip pain, restore function, and improve patients’ overall health status. When coxarthrosis progresses to its advanced stage (Stage III), patients experience persistent pain, rendering conservative treatments, such as nonsteroidal anti-inflammatory drugs (NSAIDs), neuromuscular relaxants, cartilage protectors, and other drugs, largely ineffective. Coxarthrosis can have traumatic or non-traumatic etiology, while posttraumatic osteoarthritis is associated with a higher incidence of intraoperative and postoperative complications, including infection, instability, periprosthetic fractures, and decreased survival [1-3].

A multitude of patient characteristics and related prosthetic technical factors influence the short—and long-term outcomes of THA and, subsequently, the overall longevity of a THA prosthesis. Typically, a THA prosthesis has a lifespan of approximately 15 to 20 years [4,5].

Different designs and geometry are available for the prosthetic components, with either cemented or uncemented femoral stem options. However, contemporary THA techniques have evolved into press-fit femoral and acetabular components [6,7]. Preoperative evaluation, including a thorough clinical examination and imaging studies, such as pelvic X-rays, is essential for accurately assessing the stage of arthrosis and planning the surgical intervention. This planning is critical for the success of the procedure. Patients should also be informed of potential complications, such as deep vein thrombosis and pulmonary thromboembolism, infection, limb length discrepancies, lameness, dislocation, nerve damage or bleeding, fractures, aseptic loosening, inflammation, or wound complications [8-10].

Postoperative pain management is a key factor in recovery and rehabilitation. Pain intensity is inherently subjective and can only be fully appreciated by the individual experiencing it. Hence, pain control after surgery can make a difference in recovery time. However, in medical practice, there is a need to objectify its intensity, and for this purpose, various uni- or multi-dimensional assessment tools were proposed [11,12].

The Visual Analogue Scale (VAS) was published by the World Health Organization (WHO) in 1980 to standardize analgesic therapy according to pain intensity. It is the most used tool for assessing pain intensity in clinical practice. Pain levels are classified as mild (VAS < 4), moderate (VAS 4–6), and severe (VAS ≥ 7). Based on this classification, the WHO recommends three stages of analgesic therapy: Stage I – non-opioid analgesics (e.g., NSAIDs, paracetamol); Stage II – weak opioid analgesics; and Stage III – strong opioid analgesics [13-15].

In this context, our study aimed to assess pain intensity in patients who underwent THA, employing the VAS. By categorizing pain levels based on diagnosis and surgical procedure, we seek to facilitate the appropriate selection of analgesic treatments tailored to patient needs. Pain assessment using VAS is important because it offers a high degree of resolution, or, in other words, the option of very fine nuances of judgment for practical application [16].

## MATERIAL AND METHODS

### Participants

The study was conducted between January 2022 and November 2023, enrolling 203 participants admitted to the Orthopedics Department of the Emergency County Clinical Hospital, Oradea, Romania. The research was conducted in compliance with the Declaration of the World Medical Association of Helsinki. Participation in the study was voluntary, and written informed consent was obtained from all participants to collect information and process data accurately. All participants underwent THA due to coxarthrosis, either unilateral or bilateral.

The age of participants ranged from 24 to 90 years (mean ± standard deviation: 58.44 ± 17.41 years). Among the participants, 45.3% (*n* = 92) were women, and 54.7% (*n* = 111) were men. Participants were categorized according to diagnosis into four relatively homogeneous samples, namely operated left coxarthrosis (*n* = 48; 23.6%), operated right coxarthrosis (*n* = 55; 27.1%), bilateral coxarthrosis operated unilaterally (*n* = 57; 28.1%) and bilateral coxarthrosis operated bilaterally (*n* = 43; 21.2%).

The inclusion criteria were adults who underwent THA and had their surgical procedures performed by the same surgeons. Smokers, patients with a recent history of SARS-CoV-2, patients with multiple comorbidities (including malignant tumors or organ failure), patients who refused to participate in the study, and patients who refused to sign the informed consent were excluded.

Respondents were asked to self-evaluate their post-surgical pain 24 hours post THA, using VAS, by selecting the rating corresponding to their opinion (Supplementary material S1). The collected data were processed using the statistical program IBM SPSS software version 22.0 (IBM Corp., Armonk, NY). Frequency analysis was conducted using the chi-square (χ^2^) test to evaluate the distribution of pain intensity across the four diagnostic groups.

Supplementary Material

## RESULTS

### Sociodemographic characteristics

The demographic data of participants, including gender and age distribution across the diagnostic categories, are presented in Table 1.

**Table 1:** Demographic characteristics of the participants

Diagnostic group	*n* (%)	Age range	Mean age ± SD
Operated left coxarthrosis	48 (23.6%)	28–78	47.77 ± 12.36
Operated right coxarthrosis	55 (27.1%)	24–90	50.87 ± 16.03
Bilateral coxarthrosis unilateral surgery	57 (28.1%)	33–89	62.85 ± 15.81
Bilateral coxarthrosis bilateral surgery	43 (21.2%)	32–89	74.2 ± 11.68
**Gender**			
Women	92 (45.3%)		
Men	111 (54.7%)		
Total	203		

### Assessment of postoperative pain

**Figure 1:**
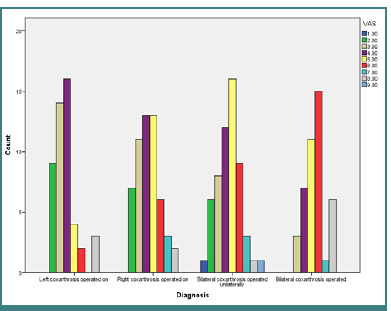
Postoperative pain distribution by diagnosis

As the VAS scale is commonly employed for pain assessment, we decided to use this scale with self-report properties in our study by analyzing the relationship between diagnosis, surgical procedures, type of hip prosthesis, and VAS. As shown in Figure 1, participants with operated left or right coxarthrosis reported irritation or mild pain that could distract attention, requiring the administration of moderate-intensity analgesics. In the group of participants with unilateral operated coxarthrosis, the pain could not be ignored for more than 30 minutes, where the therapeutic pipeline requires the administration of moderate-intensity analgesics. However, participants with bilateral coxarthrosis required the administration of strong analgesics to alleviate pain so that social activities or work could be continued.

**Figure 2:**
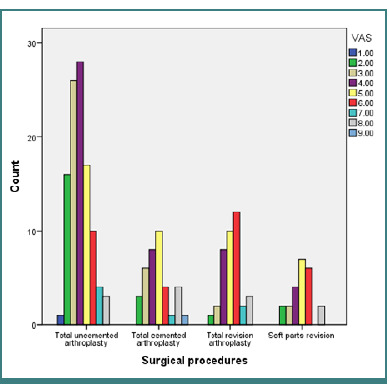
Postoperative pain distribution according to surgical procedures

In Figure 2, we present the observed frequencies of pain distribution in the postoperative phase based on surgical procedures. Participants undergoing uncemented total hip arthroplasty reported pain levels ranging from irritation to pain that could be ignored, requiring moderate-intensity analgesic. The χ^2^ test revealed significant differences (χ^2^ (24, 203) = 37.192; *P* = .04), primarily in the uncemented total arthroplasty group.

The relationship between postoperative pain and the type of hip prosthesis was further analyzed, considering preoperative health conditions and other variables. Similar to the results in Figure 2, participants with uncemented total hip prostheses reported the highest frequency of mild pain that could be ignored for short periods, requiring moderate-intensity analgesics.

**Figure 3:**
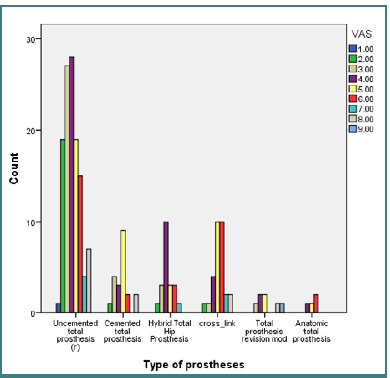
Postoperative pain according to the type of hip prosthesis

Conversely, participants with cross-link-type prostheses experienced higher pain levels that could not be ignored, necessitating strong analgesics (Figure 3). The χ^2^ test confirmed significant differences (χ^2^ (40, *n* = 203) = 73.959, *P* = 0.001), suggesting that pain values on the VAS scale for this group frequently reached levels 5 or 6. These levels indicate relatively tolerable pain, manageable with a well-planned analgesic regimen.

## DISCUSSION

Coxarthrosis is a condition with a long-term evolution, the symptoms evolving, increasing in frequency, and becoming disabling for the patient in general and for patients with advanced age in particular [7].

Preoperative risk factors that were consistently predictive of greater postoperative pain after THA were summarized in a recent systematic review developed by Zhang *et al*. [17]. This includes preoperative body mass index (BMI), preoperative pain and hip function, age, sex, radiographic severity of arthritis, socioeconomic status and race/ethnicity, and preoperative comorbidities. According to this study, the most significant associations were found between poor pain outcomes and the female sex, high preoperative pain or low function, and various medical or psychiatric comorbidities.

Although some patients continue to experience hip pain after elective surgery, it was reported that women experienced greater pain relief than men postoperatively. Still, they exhibited lower satisfaction with the surgical procedure one-year post-THA [18].

Pain is a strong signal of decreased functionality and decreased quality of life. Based on our previous study [19], it was highlighted that coxarthrosis etiology influences the patient’s quality of life in the preoperative and postoperative phase of total hip arthroplasty, the overall results indicating that the traumatic group displayed more favorable post-surgery evolution (including a lower level of pain), and higher autonomy compared to the non-traumatic one. Moreover, physical functioning was more affected in participants with traumatic coxarthrosis, and fatigue was specific to nontraumatic coxarthrosis. Emotional well-being and social functioning were high in patients with traumatic coxarthrosis, probably because they have not experienced emotional erosion and alienation compared to people with non-traumatic coxarthrosis [19].

In the present study, the analysis of treatments administrated according to the diagnosis revealed that antibiotic therapy and thromboprophylaxis were commonly employed in coxarthrosis, as well as anticoagulants. The extended treatment scheme was specific to unilateral and bilateral operated coxarthrosis. While postoperative medical prescriptions are often necessary, palliative treatments were predominantly used for both unilateral and bilateral surgical cases. We noticed a favorable postoperative evolution in the case of unilaterally operated coxarthrosis, while in the case of bilaterally operated coxarthrosis, the evolution was less favorable, being associated with the age of the patients and the severity of the disease.

The emotional state and the acceptance of the functional state related to quality of life play a special role in evaluating patient quality of life in patients with coxarthrosis and chronic osteoarticular degenerative diseases. These diseases represent a public health problem due to their duration, family, social, economic, and medical implications [20].

Based on the previously reported data in the literature [21,22], we aimed to investigate the functionality outcomes and post-surgical pain in coxarthrosis. We noticed that people with operated unilateral coxarthrosis often experience irritation and pain that can be distracting, requiring the administration of moderate-intensity analgesics. For people with unilaterally operated coxarthrosis, the pain cannot be ignored for more than 30 minutes, as moderate-intensity analgesics are required, while for people with bilaterally operated coxarthrosis, the administration of strong analgesics is required to alleviate pain and increase functionality.

Additionally, we analyzed the relationship between postoperative pain and the type of surgical procedure performed. In cases where uncemented total arthroplasty was used, patients often experienced irritation and tolerable pain, which typically required moderate-intensity analgesics for relief. However, the use of cross-link type prostheses was associated with more severe pain that could not be ignored, necessitating the administration of strong analgesics to manage discomfort effectively.

Our findings agree that patient satisfaction plays a crucial role in modern healthcare systems. However, assessing satisfaction remains challenging due to its multifactorial nature and the absence of a standardized measurement method. Despite this, the VAS has been demonstrated to be a valuable tool for evaluating patient satisfaction following THA, complementing both subjective and objective outcome measures.

Our study has several limitations. Due to its observational and retrospective character, it lacks reliable baseline data and a control group, which limits our ability to assess long-term outcomes. A longitudinal design would provide a more comprehensive understanding of the healing process over time. Additionally, the study population was drawn from a single private clinic, which may limit the generalizability of the findings. While scales such as the VAS are widely used due to their simplicity, they do not fully capture the range of issues associated with traumatic or non-traumatic coxarthrosis.

Furthermore, not all relevant scales are available in Romanian or have been adequately validated. These findings align with Henry and Foster's observation that, although numerous observational instruments have been developed over the past two decades to measure pain levels, none are specifically tailored to chronic pain conditions [23]. For future research, we plan to conduct a more comprehensive study involving longitudinal comparisons of pain levels before and after surgery, as VAS scores may vary depending on the analgesics patients receive.

## CONCLUSION

Based on the VAS assessment and statistical frequency analysis using the χ^2^ test, we concluded that patients with unilaterally operated coxarthrosis experienced pain that could not be ignored for more than 30 minutes, necessitating the use of moderate-intensity analgesics. In contrast, patients with bilaterally operated coxarthrosis required strong analgesics to manage pain and improve functionality. Regarding the surgical procedure, the highest frequency of tolerable irritation or pain, which could be ignored for short periods and required moderate-intensity analgesics, was observed in patients who received uncemented total hip prostheses. However, when cross-link type prostheses were used, post-surgical pain was significantly more intense, necessitating the administration of strong analgesics to provide relief.
