# Supplementary material for: Assessment of pain intensity after total hip arthroplasty using the Visual Analogue Scale (VAS)
Source: J Med Life. 2024 Dec;17(12):1049–53. doi: 10.25122/jml-2024-0362 (PMC11771834; doi:10.25122/jml-2024-0362)
Supplement: Supplementary file 1 [file JMedLife-17-1049-s001.pdf]

Supplementary Material

Table S1. Visual Analogue Scale (VAS)

SCORE	SYMPTOMATOLOGY	THERAPEUTIC CONDUCT
0	No pain	No medication required
1	Minor irritation occasionally; minor painful flare-ups	Medication as needed
2	Occasional irritation; strong painful attacks	Medication as needed
3	Irritation sufficient to distract from activities	Moderate-intensity analgesics
4	Pain can be ignored if engaged in work but remains distracting	Moderate-intensity analgesics
5	Pain cannot be ignored for more than 30 minutes	Moderate-intensity analgesics
6	Pain cannot be ignored at all, though work or social activities can continue	Strong analgesics
7	Pain interferes with concentration and sleep; work continues with effort	Strong analgesics
8	Severe limitation in physical activity; reading and conversation require effort	Strong analgesics
9	Inability to speak; uncontrollable crying or moaning, near delirium	The strongest pain relievers are partially effective
10	Incontinence; pain severe enough to be life-threatening	The strongest pain relievers are only partially effective

© 2024 by the authors. This article is an open access article distributed under the terms and conditions of the Creative Commons Attribution (CC BY 4.0) license.
